# Effect of Chlorhexidine Solution on the Tensile Bond Strength and Hardness of Two Soft Liners With Denture Base Resin: An In-Vitro Comparative Study

**DOI:** 10.7759/cureus.94446

**Published:** 2025-10-13

**Authors:** Deepak Kumar Samal, Tapan K Patro, Angurbala Dhal, Lokanath Garhnayak, Ullash Kumar

**Affiliations:** 1 Prosthodontics, Crown and Bridge, Srirama Chandra Bhanja (SCB) Dental College and Hospital, Cuttack, IND

**Keywords:** chlorhexidine gluconate, denture, denture cleanser, hardness, resilient denture liner, soft liner, tensile bond strength, tensile strength

## Abstract

Background

Edentulism presents significant functional, nutritional, and psychological challenges, often requiring complete denture rehabilitation. Soft denture liners are used to enhance comfort and fit, and functionality of existing dentures, especially in patients with compromised oral tissues. However, the longevity of their performance is hindered by the deterioration of tensile bond strength and material softness. Chlorhexidine gluconate solution, recommended as a denture cleanser, may alter these properties.

Objective

To evaluate the effect of 0.2% chlorhexidine gluconate solution (HEXIDINE -ICPA Health Products Ltd, Mumbai, India) on the tensile bond strength and Shore A hardness values of two types of denture liners: acrylic-based, Permasoft (Perma Laboratories, Broadview Heights, OH, USA), and silicone-based Ufi Gel P (VOCO GmbH, Cuxhaven, Germany), bonded to heat-cured polymethyl methacrylate (PMMA) denture base resin (AcryPol R, Ruthinium, Dental Manufacturing S.P.A., Badia Polesine, Italy). This study investigated whether the daily use of chlorhexidine solution alters the mechanical properties of these commonly used denture liners.

Materials and methods

Seventy-eight specimens were prepared to assess the effect of 0.2% chlorhexidine gluconate on the tensile bond strength and Shore A hardness of two soft denture liners: acrylic-based, Permasoft, and silicone-based, Ufi Gel P, bonded to heat-cured PMMA resin. For tensile strength testing, fifty-two specimens were fabricated by bonding two PMMA blocks (40 × 10 × 10 mm) with a 3 mm thick liner. For hardness testing, twenty-six cylindrical specimens (6 mm height, 10 mm diameter) were prepared. Specimens were divided into two groups for immersion in distilled water as a control and in 0.2% chlorhexidine gluconate solution. Tensile bond strength was measured using a universal testing machine. Failure modes were classified as adhesive, cohesive, or mixed. Hardness was evaluated using a Shore A digital durometer, with five readings per specimen.

Results

Permasoft exhibited significantly higher tensile bond strength (mean: 0.72 MPa) compared to Ufi Gel P (mean: 0.60 MPa). Shore A hardness values were also greater for Permasoft (mean: 45.5) than Ufi Gel P (mean: 36.1). Specimens immersed in distilled water showed higher tensile bond strength than those treated with 0.2% chlorhexidine gluconate (P = 0.001). Conversely, Shore A hardness increased following chlorhexidine immersion for both liners, with Permasoft reaching the highest value (49.3). Two-way analysis of variance (ANOVA) revealed significant main effects of material type and solution on both tensile bond strength and hardness (P = 0.001).

Conclusions

Both Permasoft and Ufi Gel P demonstrated clinically acceptable tensile bond strength with PMMA denture base resin. However, daily immersion in 0.2% chlorhexidine gluconate solution significantly reduced tensile bond strength and increased Shore A hardness in both materials compared to distilled water. Ufi Gel P showed better compatibility with chlorhexidine solution than Permasoft, under disinfectant exposure. While chemical denture cleansers are effective for oral hygiene, their impact on material properties should be carefully considered in clinical practice.

## Introduction

Alterations in underlying denture base tissue due to ageing, metabolic problems, defective denture use may cause some degree of discomfort and lesions, or else aggravate existing lesions in the mucosal lining. Conventional complete dentures fail to satisfy patients with traumatised mucosa, severe undercuts in the underlying bone, ridge atrophy, or bruxism [1,2]. 

To combat this problem, the simplest and first approach is the application of denture liners on the intaglio surface of the denture base [3]. Denture relining materials are commonly used to improve the fit between the denture and the mucous membrane [4]. These materials are recommended for clinical situations characterised by irregular bone resorption, thin atrophic mucosa, and the presence of severe bony undercuts because they act as a cushion and provide an even distribution of functional load onto the stress-bearing mucosa [5]. Studies have shown that edentulous patients reported greater comfort when their dentures were lined with soft liners and also increased the patient’s masticatory performance, biting force, and improved the chewing rhythm [6-8].

The optimum thickness for a liner is approximately 2.5 to 3 mm, which is needed to provide good shock absorption [9]. Ideal properties of resilient liners include resiliency, which is desired over a long period of time, and a durable bond to the denture base [10].

One of the problems with denture liners is the failure of the bond between the resilient denture liner and the denture base resin [11]. A successful relining depends on the bond strength between the liner and the resin base [12]. Tensile bond strength testing is one of the various methods for assessing the bond strength of soft liners. Durability of softness during clinical use is a desirable feature of a soft liner. Any increase in hardness can worsen the distribution of the masticatory load and lower the absorption of elastic energy, which is transmitted onto the mucosal membrane under dentures, thereby exacerbating the clinical problems experienced by patients [13].

Drawbacks of denture relining materials include an increase in hardness, colour changes, porosity, microbial colonisation, especially Candida albicans, insufficient tear strength, and poor bond strength [14]. To mitigate these problems, maintaining effective denture plaque control through both mechanical and chemical methods is vital. While mechanical cleansing can be an effective approach for traditional dentures, it is not recommended for soft denture liners, as the abrasive action can cause damage to these delicate, resilient materials. As a result, chemical cleansing employing specially formulated denture cleaners has proven to be the most effective strategy for controlling plaque buildup on dental liners [15]. However, such procedures may lead to changes in the properties of the soft liners.

Chlorhexidine gluconate is an antiseptic agent with a broad spectrum of antimicrobial and antifungal properties and is therefore commonly used in mouthrinses and denture cleansers [16]. Permasoft is an acrylic-based permanent soft liner, whereas Ufi Gel P is a silicone-based permanent soft liner.

Hence, the study of tensile bond strength and hardness between the soft liners and the polymethyl methacrylate (PMMA) denture base material is an essential requirement for the durability of their use in situ. Many studies have been reported on bond strength, roughness, solubility, and hardness of different soft liners; however, there is inadequate literature on the effect of chlorhexidine gluconate solution on the tensile bond strength and hardness of denture liners. Hence, this study was planned to study the effect of chlorhexidine gluconate solution on denture liners. The null hypothesis was that there would be no difference in tensile bond strength and Shore hardness between the two types of soft liners when immersed separately in distilled water and 0.2% chlorhexidine gluconate solution.

## Materials and methods

This in-vitro study was conducted at the P.G. Department of Prosthodontics and Crown & Bridge, Srirama Chandra Bhanja (SCB) Dental College and Hospital, Cuttack, Odisha, and the Laboratory for Advanced Research in Polymeric Materials (LARPM), Council of Scientific and Industrial Research - Central Institute of Petrochemicals Engineering & Technology (CIPET), Bhubaneswar, Odisha. Ethical clearance was obtained from the Institutional Ethical Committee (Reg. No. IEC/SCBDCH/-3-/2023). The study was carried out over a period of two years.

Sample Size estimation

The sample size was calculated based on a previous study conducted by Mese et al. [9]. The pooled mean was 0.65 MPa with a standard deviation of 0.12 MPa, yielding an effect size of 1.08. With α = 0.05 and power = 0.80, a minimum of thirteen specimens per group was calculated. To accommodate two liner types and two immersion conditions, fifty-two specimens were prepared for tensile bond strength testing. For Shore A hardness, the pooled mean and standard deviation (SD) were 40.2 and 6.5, respectively, yielding an effect size of 1.0; twenty-six specimens were fabricated. 

The specifications of the specimens were as follows: 1) For tensile bond strength testing - Fifty-two specimens cuboidal blocks of heat-cured PMMA denture base material (AcryPol R, Ruthinium, Dental Manufacturing S.P.A., Badia Polesine, Italy) of dimension 40 mm × 10 mm × 10 mm attached to a liner of dimension 3 mm x 10 mm x 10 mm. 2) For Shore A hardness testing - Twenty-six cylindrical specimens of acrylic-based liner (Permasoft, Perma Laboratories, Broadview Heights, OH, USA) and silicone-based liner (Ufi Gel P, VOCO GmbH, Cuxhaven, Germany) of height 6 mm and diameter of 10 mm.

For tensile bond strength testing

**Figure 1:**
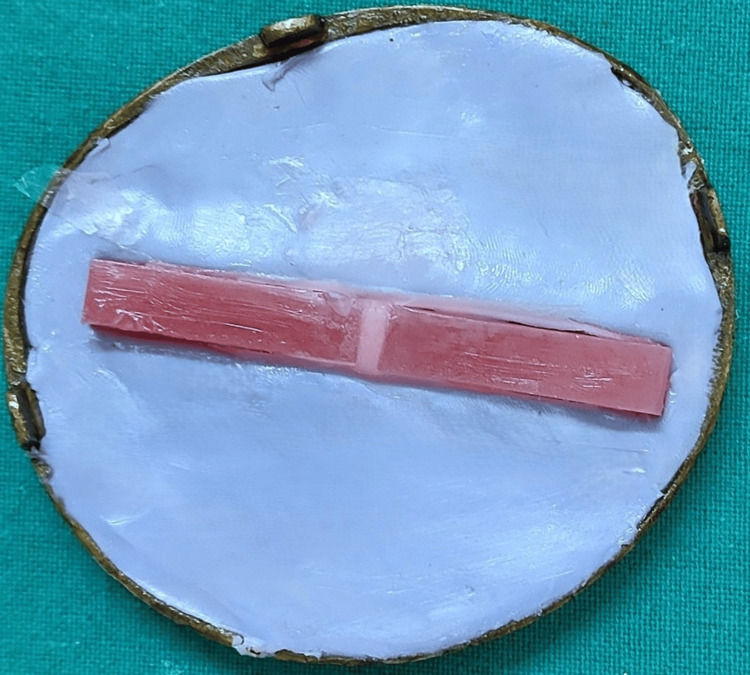
Specimen with excess liner flash after polymerisation

After fabrication, PMMA blocks underwent ultrasonic cleaning using distilled water to eliminate surface impurities, followed by drying with compressed air. The two PMMA blocks were placed in the flask, and the liner was applied according to the respective manufacturer’s instructions. Then the flask was closed and the liner was allowed to polymerise. Following the recovery of the specimen, the excess liner flash was cut using a sharp blade (Figure 1). As a result, a soft liner was placed between two PMMA blocks to obtain the final specimens.

**Figure 2:**
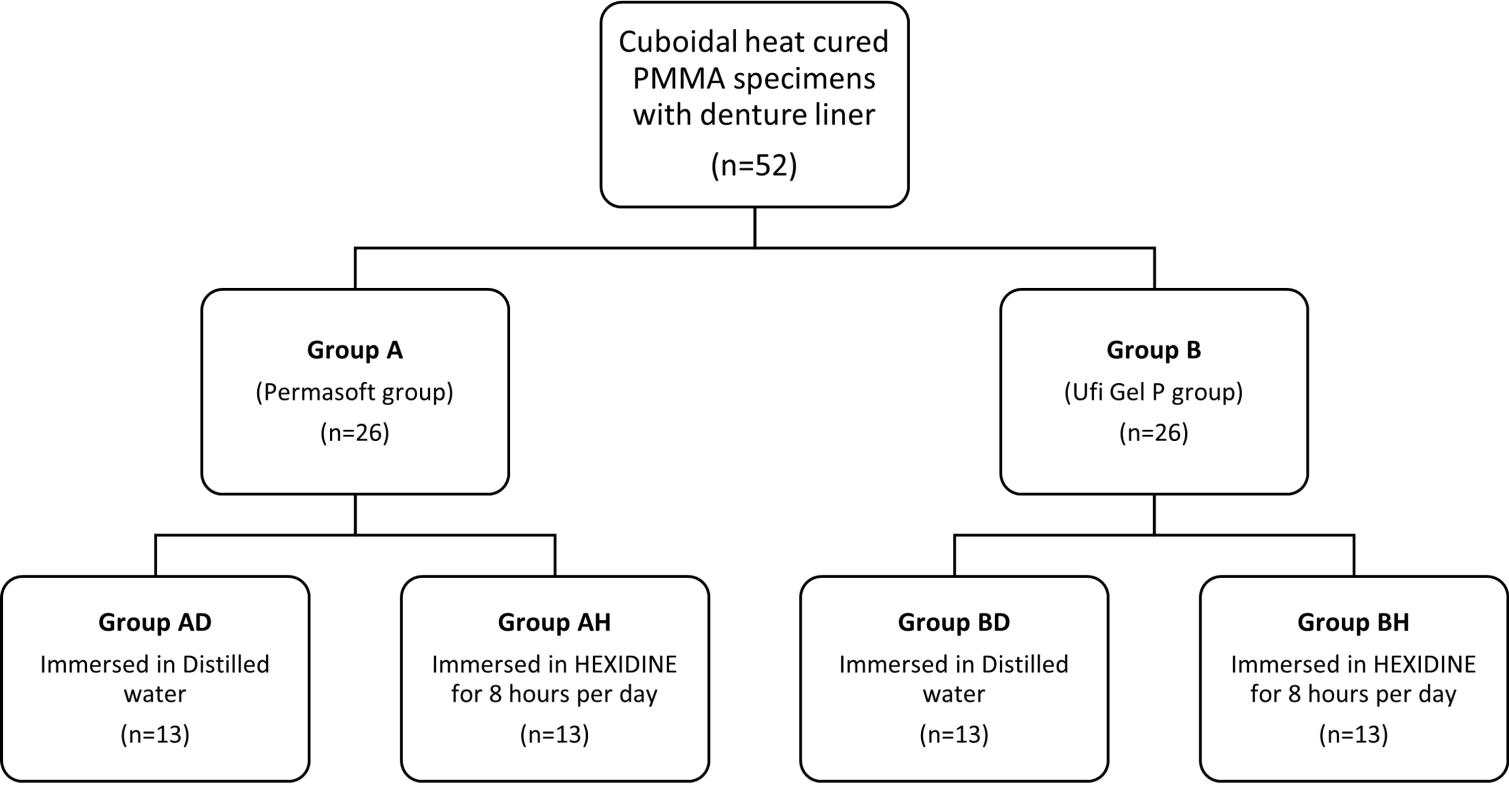
Study design flow chart for evaluation of tensile bond strength

Thirteen specimens of each liner group were immersed in distilled water and kept at room temperature for thirty days before testing. The remaining thirteen specimens of each liner group were immersed in 0.2% chlorhexidine gluconate solution (HEXIDINE, ICPA Health Products Ltd, Mumbai, India) for eight hours once per day to simulate the clinical scenario of daily disinfection and storage of the denture at night. After eight hours, they were rinsed with distilled water and immersed in it at room temperature for the next sixteen hours. This procedure was repeated for thirty days. The study design flow chart is depicted in Figure 2.

**Figure 3:**
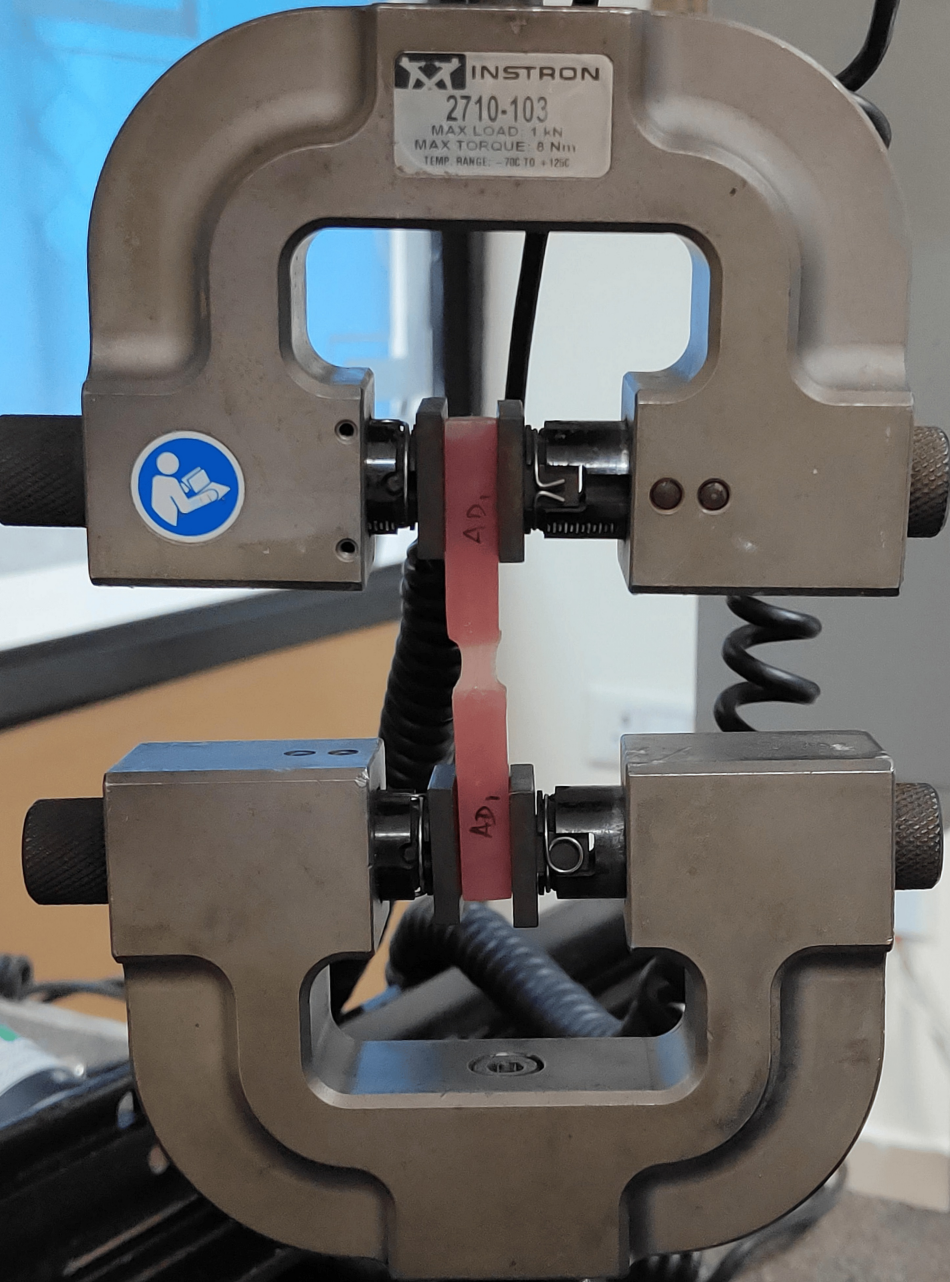
Specimen under tensile load in Universal testing machine

After that, all specimens were taken from solutions and rinsed with running water before undergoing testing. All the specimens were aligned in a Universal testing machine (Instron, Norwood, MA, USA). Testing was conducted at 21 ± 1 °C. The specimens were pulled with a crosshead speed of 5 mm/min (Figure 3). For every specimen, the highest tensile stress before failure was noted. The test results were documented in MPa.

Failure mode evaluation

The type of failure was carefully evaluated through visual inspection and categorised as 1) adhesive: indicated separation occurring at the liner-PMMA interface, 2) cohesive: indicated failure within the liner material, and 3) mixed: indicated failure at both the liner-PMMA interface and within the liner.

Hardness testing

Following fabrication, the specimens designated for hardness testing were stored under the exact conditions as those prepared for tensile strength evaluation. After that, all specimens were taken from the two solutions and rinsed with running water before undergoing testing. A Shore A digital durometer (PosiTector® SHD, DeFelsko Corporation, NY, USA) was used for hardness testing. The final value of the hardness depended on the penetration depth of the indenter. For each specimen, five readings were obtained.

All tests were conducted by the same operator to standardise testing conditions and ensure uniformity. Standardised procedures for testing have been defined by the respective ISO and ASTM D2240 standards [17,18].

Statistical analysis

The data was analysed utilising SPSS for Windows version. 26.0, (IBM Corp., Armonk, NY). Comparisons of tensile bond strength and Shore A hardness scores were made between different types of soft liners and various solutions through the use of unpaired t-tests. Additionally, a two-way analysis of variance (ANOVA) was conducted to assess the impact of groups and solutions on both Tensile bond strength and Shore A hardness. Chi-square test was performed to evaluate the association between liner type (Permasoft vs Ufi Gel P) and observed failure modes (adhesive, cohesive, mixed). Significance level was established at P < 0.05.

## Results

It was found that Permasoft had a higher tensile bond strength when compared to Ufi Gel P. The difference in mean tensile bond strength between the groups was statistically significant (P = 0.001) (Table 1).

**Table 1:** Comparison of mean tensile bond strength of Permasoft and Ufi Gel P SD-standard deviation; **statistically significant using the unpaired t-test

		Number	Mean ± SD (MPa)	t	P value
Tensile Bond Strength	Permasoft	26	0.72 ± 0.13	3.4	P = 0.001**
	Ufi Gel P	26	0.6 ± 0.11		

**Figure 4:**
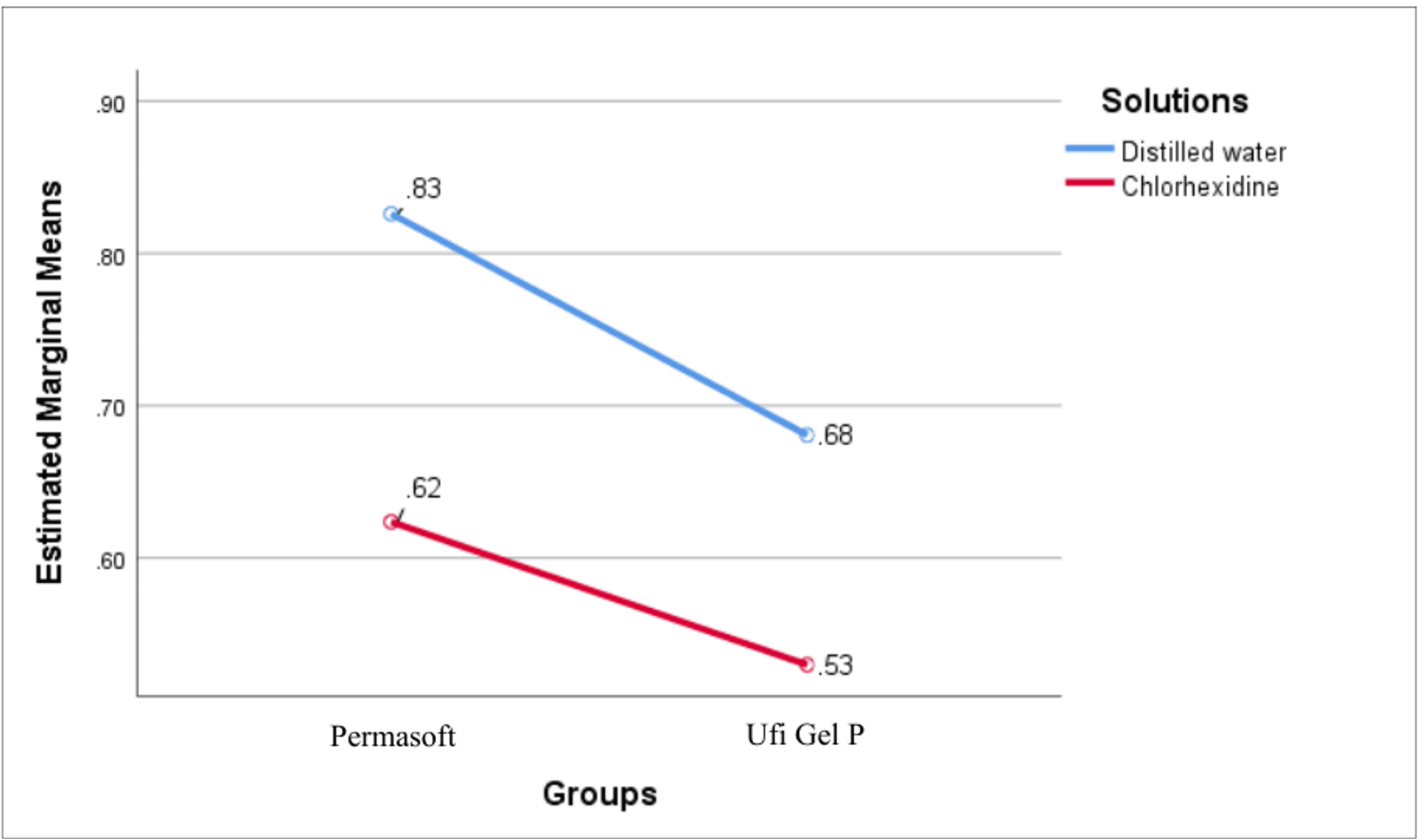
Mean tensile bond strength (in MPa) for different soft liners and solutions

When immersed in distilled water, it was found that both Permasoft and Ufi Gel P had higher tensile bond strength when compared to chlorhexidine (Figure 4). The difference in mean tensile bond strength of Permasoft and Ufi Gel P between the groups was statistically significant (P = 0.001) (Table 2).

**Table 2:** Comparison of mean tensile bond strength between distilled water and chlorhexidine for Permasoft and Ufi Gel P SD-standard deviation; **statistically significant using unpaired t-test

		Number	Mean ± SD (MPa)	t	P value
Permasoft	Distilled Water	13	0.826 ± 0.07	5.6	P = 0.001**
	Chlorhexidine	13	0.623 ± 0.106		
Ufi Gel P	Distilled Water	13	0.68 ± 0.08	4.6	P = 0.001**
	Chlorhexidine	13	0.529 ± 0.08		

The results also showed that Permasoft had a higher Shore A hardness value when compared to Ufi Gel P. The difference in mean Shore A hardness values between the groups was statistically significant (P = 0.001) (Table 3).

**Table 3:** Comparison of mean Shore A hardness values of Permasoft and Ufi Gel P SD-standard deviation; **statistically significant using the unpaired t-test

		Number	Mean ± SD	t	P value
Shore A Hardness	Permasoft	26	45.5 ± 4.2	9.6	P = 0.001**
	Ufi Gel P	26	36.1 ± 2.6		

**Figure 5:**
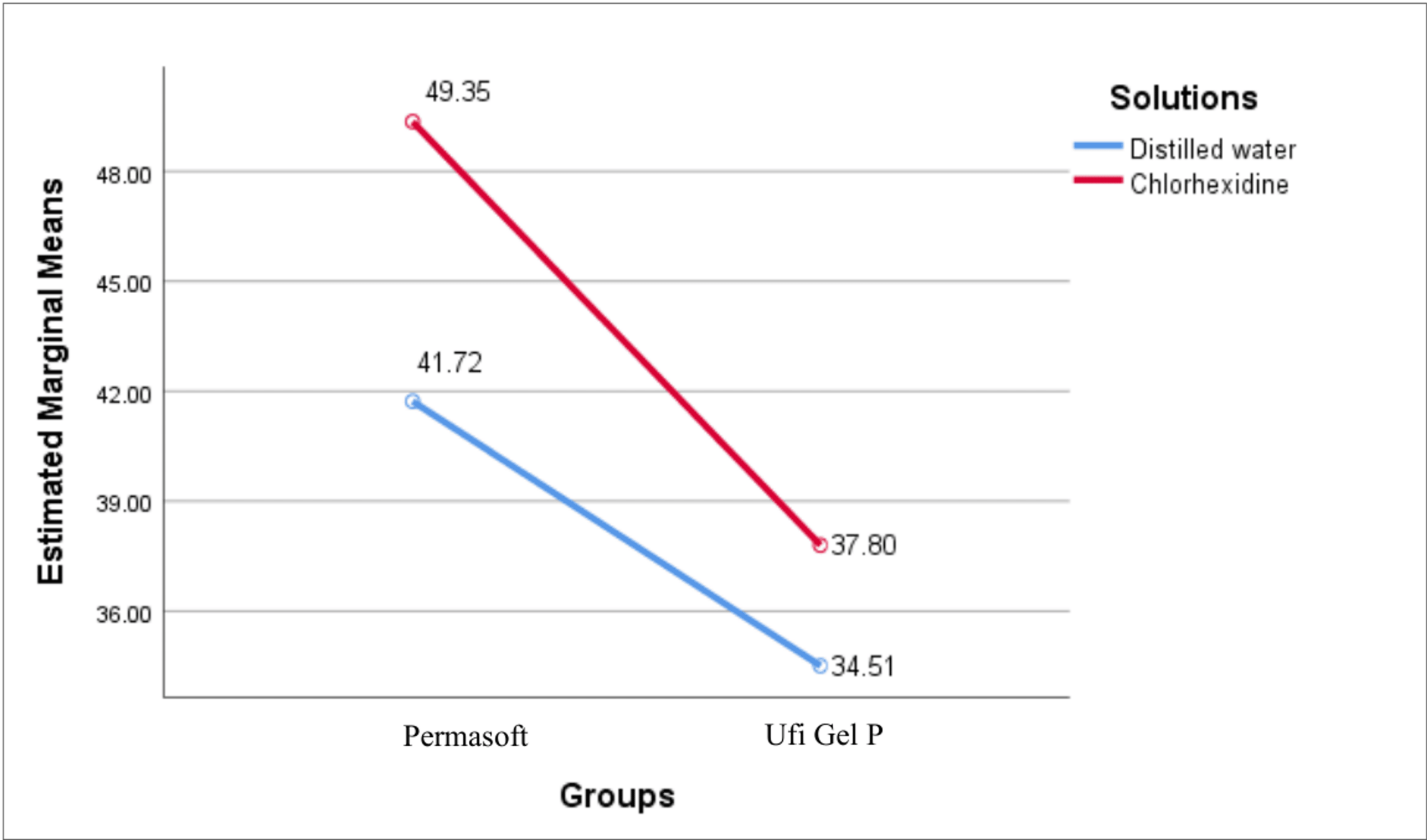
Mean Shore A hardness values for different soft liners and solutions

The mean Shore A hardness values of both Permasoft and Ufi Gel P when immersed in chlorhexidine were higher when compared to distilled water (Figure 5). The difference in mean Shore A hardness values of Permasoft and Ufi Gel P between the groups was statistically significant (P = 0.001) (Table 4).

**Table 4:** Comparison of mean Shore A hardness values between distilled water and chlorhexidine for Permasoft and Ufi Gel P SD-standard deviation; **statistically significant using unpaired t-test

		Number	Mean ± SD	t	P value
Permasoft	Distilled Water	13	41.7 ± 1.6	-11.8	P = 0.001**
	Chlorhexidine	13	49.3 ± 1.6		
Ufi Gel P	Distilled Water	13	34.5 ± 1.8	-4.06	P = 0.001**
	Chlorhexidine	13	37.8 ± 2.2		

Two-way ANOVA tests revealed statistically significant main effects of material type and immersion medium on both tensile bond strength and Shore A hardness (P = 0.001). Permasoft exhibited higher tensile bond strength and Shore A hardness compared to Ufi Gel P. Specimens immersed in distilled water demonstrated greater tensile bond strength, whereas chlorhexidine solution exposure increased Shore A hardness values. The interaction effect for tensile bond strength was not significant (P = 0.29), indicating a uniform reduction across Permasoft and Ufi Gel P (Table 5). In contrast, a significant interaction was observed for Shore A hardness (P = 0.001), with Permasoft showing a more pronounced response to chlorhexidine solution exposure (Table 6).

**Table 5:** Comparison of different materials (Permasoft and Ufi Gel P) and solutions (distilled water & chlorhexidine solution) regarding tensile bond strength by two-way ANOVA **statistically significant, ***statistically not significant

	Type III Sum of Squares	Df	Mean Square	F	P value
Groups	0.186	1	0.186	24.33	P = 0.001**
Solutions	0.405	1	0.405	53.01	P = 0.001**
Groups x Solutions	0.009	1	0.009	1.12	P = 0.29***

**Table 6:** Comparison of different materials (Permasoft & Ufi Gel P) and solutions (distilled water & chlorhexidine solution) regarding Shore A hardness scores by two-way ANOVA **statistically significant

	Type III Sum of Squares	df	Mean Square	F	P value
Groups	1144.9	1	1144.9	327.8	P = 0.001**
Solutions	387.7	1	387.7	111.04	P = 0.001**
Groups x Solutions	61.1	1	61.1	17.5	P = 0.001**

**Figure 6:**
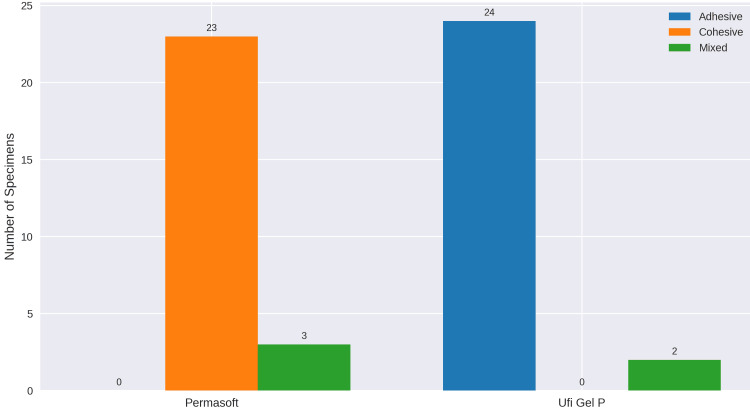
Distribution of failure modes

Chi-square analysis revealed a statistically significant association between liner type and failure mode (χ² = 47.2, df = 2, P < 0.0001). Permasoft specimens predominantly exhibited cohesive failures, while Ufi Gel P specimens showed adhesive failures (Figure 6).

## Discussion

Resilient denture liners offer a cost-effective and minimally invasive approach for prolonging the functional lifespan of existing prostheses. Materials intended for the relining of removable dentures must exhibit several key characteristics that encompass their compatibility with oral tissues, ability to recover from deformation, resistance to changes in shape and colour, endurance against surface wear, and the strength of adhesion to the denture base [19]. This study investigated two key characteristics of denture liners: tensile bond strength with the denture base resin and Shore A hardness.

The bonding of soft denture liners with the denture base has been assessed using tensile tests, shear tests, and peel tests. McCabe et al. suggested that using both peel and tensile test methods is relevant and effective for examining the bonding and debonding characteristics of soft material polymers [20]. Al-Athel et al. indicated that the tensile bond strength test is an appropriate method for assessing the bond strength of soft liners [21]. In this study, the bond strength between soft denture liners and PMMA denture bases was evaluated using a tensile test, as outlined by the American Society for Testing and Materials [18].

Neglecting proper denture care can significantly diminish the lifespan of dentures and lead to an alarming buildup of plaque. This accumulation not only affects the hygiene of the dentures but can also pose risks to the wearer’s oral health, creating complications that may require additional dental intervention. Chlorhexidine gluconate has been widely used as an oral mouthrinse with varying concentrations between 0.2-4%. Haydari et al. showed that mouthwash containing 0.2% chlorhexidine gluconate had a significantly better effect in preventing dental plaque than the 0.12% and 0.06% solutions [22]. So, in this study, a 0.2% chlorhexidine solution (HEXIDINE) was designated as the experimental group. In contrast, distilled water was employed as the control group, providing a neutral baseline for comparison.

The results showed that there was a significant difference in tensile bond strength and hardness between Permasoft and Ufi Gel P when immersed separately in distilled water and chlorhexidine gluconate solution (0.2%) (HEXIDINE). Thus, the null hypothesis was rejected.

Permasoft had a significantly higher tensile bond strength when compared to Ufi Gel P, both when immersed in distilled water and HEXIDINE. Similar studies were also conducted by Mese et al., in which they found that the chemical composition of liners affected the tensile bond strength [9]. This was due to the stronger bond strength between the liner and PMMA material compared to the bond strength within the liner. These results can be attributed to the similar chemical composition of the PMMA denture base and Permasoft, resulting in chemical adhesion, whereas in the case of Ufi Gel P, the bond with the PMMA denture base was mechanical in nature. These results were in contrast to the results obtained by El-Hadary et al. [23]. They found out that the silicone-based liner showed higher tensile bond strength than Permasoft. They attributed it to the improved adhesive bonding systems used with silicone-based soft liners.

Moreover, HEXIDINE affected the tensile bond strength adversely, as immersion in HEXIDINE resulted in a measurable reduction in the tensile bond strength of both the liner groups. The percentage bond strength reduction when submerged in HEXIDINE was greater for the Permasoft group compared to the Ufi Gel P group. However, the mean tensile bond strength of both subgroups of Permasoft and Ufi Gel P was 0.72 and 0.6 MPa, respectively. These values were higher than the minimum tensile bond strength needed to be acceptable for clinical usage, which was 0.44 MPa (4.5 kg/cm²) [24,25].

**Figure 7:**
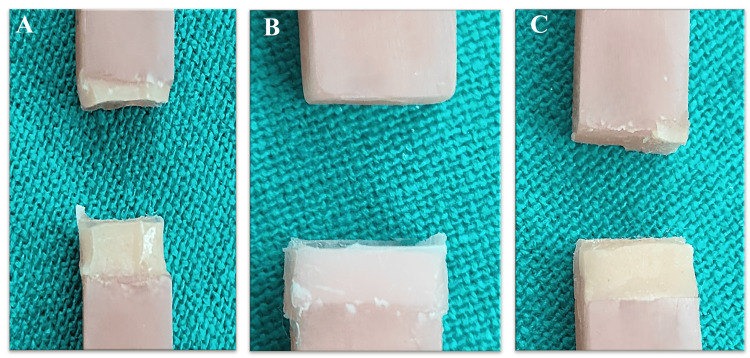
Mode of failure A-cohesive failure (Permasoft), B-adhesive failure (Ufi Gel P), C-mixed failure (Permasoft)

Regarding the failure mode, for Permasoft specimens, all failures were cohesive in nature (Figure 7A). Except for three specimens, which exhibited mixed failure (Figure 7C). This can be attributed to Permasoft’s superior bond strength, likely resulting from its similar chemistry with the PMMA denture base. Among the twenty-six Ufi Gel P specimens, most of the specimens failed adhesively (Figure 7B). While two exhibited a mixed type of failure. This was due to the lower bond strength between the liner and PMMA material in comparison to the bond strength within the liner material.

Permasoft exhibited a higher Shore A Hardness score when immersed in both distilled water and HEXIDINE, compared to Ufi Gel P, which recorded lower scores in both solutions. This was consistent with the findings of a study conducted by Pahuja et al., who found that silicone-based liners maintained their resiliency better than acrylic-based liners when immersed in different denture cleanser solutions [26].

Permasoft exhibited a greater percentage increase in Shore A hardness compared to Ufi Gel P. This finding was consistent with a study conducted by Brożek et al., who concluded that silicone-based relining materials were less affected by denture cleansers than the acrylic-based relining materials with respect to loss of elasticity [27]. This behaviour can be explained due to water absorption and the leaching of various substances, including monomers and plasticisers, into the storage solutions from acrylic-based liners.

Permasoft on immersion in HEXIDINE exhibited the highest Shore A hardness scores, whereas Ufi Gel P immersed in distilled water showed the lowest Shore A hardness scores. Similar results were obtained by Niarchou et al. [28]. This can be explained by a study conducted by Kaul et al., which showed that the release of plasticisers takes place more rapidly into ionised solutions, and chlorhexidine solution, being an ionised solution, resulted in higher Shore A hardness values than distilled water, which is free from ions [29]. Similarly, Kazanji and Watkinson reported that the presence of sodium and potassium ions, particularly, enhances the rate at which these plasticisers are released from soft liners, highlighting the significant impact these ions have on the behaviour of resilient materials [30].

Limitations of this study

One of the limitations of the present study was that the chlorhexidine solution used (HEXIDINE) contained additional components such as colouring agents and preservatives, which may have influenced the results and confounded the effect of pure chlorhexidine. Furthermore, the study did not account for variations in formulations of acrylic and silicone-based soft liners, limiting the generalizability of the findings. Artificial saliva, cyclic fatigue behaviour, and thermocycling may better simulate the oral environment with precise planning. Additional clinical studies can be conducted to further assess the findings of this research.

Clinical Implications

Daily immersion in chlorhexidine solution (0.2%) reduced bond strength and increased hardness in both liners. Denture hygiene regimens must be tailored to balance antimicrobial efficacy with liner preservation. The use of chlorhexidine solution (0.2%) as a denture cleanser should be minimised for a shorter duration of immersion to prolong the durability of denture liners. For patients with high hygiene needs, Ufi Gel P may offer better long-term performance.

## Conclusions

This study revealed that the tensile bond strength of both Permasoft and Ufi Gel P with the denture base was acceptable for clinical usage. Chlorhexidine solution (0.2%) decreased the bond strength of both Permasoft and Ufi Gel P liners and also increased the hardness of both Permasoft and Ufi Gel P liners in comparison to distilled water. Ufi Gel P exhibits superior compatibility with chlorhexidine solution compared to Permasoft, particularly in its ability to maintain resiliency. The findings also implied that chemical denture cleansers, such as chlorhexidine solution, can be used daily to clean soft denture liners. However, in the long term, they have an adverse effect on the tensile bond strength and surface hardness.
